# A Comparison of the Pitfall Trap, Winkler Extractor and Berlese Funnel for Sampling Ground-Dwelling Arthropods in Tropical Montane Cloud Forests

**DOI:** 10.1673/031.011.0128

**Published:** 2011-03-08

**Authors:** Thomas K. Sabu, Raj T. Shiju, KV. Vinod, S. Nithya

**Affiliations:** Litter Entomology Research Unit, Post Graduate & Research Department of Zoology, St. Joseph's College, Devagiri, Calicut, Kerala, India 673008

**Keywords:** forest soil-litter arthropods, Shola, the Western Ghats

## Abstract

Little is known about the ground-dwelling arthropod diversity in tropical montane cloud forests (TMCF). Due to unique habitat conditions in TMCFs with continuously wet substrates and a waterlogged forest floor along with the innate biases of the pitfall trap, Berlese funnel and Winkler extractor are certain to make it difficult to choose the most appropriate method to sample the ground-dwelling arthropods in TMCFs. Among the three methods, the Winkler extractor was the most efficient method for quantitative data and pitfall trapping for qualitative data for most groups. Inclusion of floatation method as a complementary method along with the Winkler extractor would enable a comprehensive quantitative survey of ground-dwelling arthropods. Pitfall trapping is essential for both quantitative and qualitative sampling of Diplopoda, Opiliones, Orthoptera, and Diptera. The Winkler extractor was the best quantitative method for Psocoptera, Araneae, Isopoda, and Formicidae; and the Berlese funnel was best for Collembola and Chilopoda. For larval forms of different insect orders and the Acari, all the three methods were equally effective.

## Introduction

Patches of tropical montane cloud forests (TMCF) occur in Central and South America, tropical Africa, and tropical Asia where humid mountains are frequently enveloped by tradewind-derived orographic clouds and fog in combination with convective rainfall (Still et al. 1999; Doumenge et al. 1995; Bruijnzeel 2001). Many features of these forests from vegetation morphology to nutrient budgets to solar insolation are directly or indirectly related to cloud formation. One of the most direct impacts of frequent cloud cover is cloud stripping, which is the deposition of cloud droplets through contact with vegetation and fog drip to the forest floor (fog precipitation) and the presence of moss cover (bryophytic cover) on the stem of trees (Stadtmüller 1987; Frahm and Gradstein 1991; Bruijnzeel and Proctor 1995; Holder 2006). TMCFs are often situated on mountain tops or ridge lines at various elevations, especially between 1000 and 3500 m, but under exceptional conditions they have been known to occur at low elevations as well (300–500 m asi) (Hamilton et al. 1995; Bruijnzeel 2001). TMCFs are among the most endangered of all tropical forest types and usually harbour very high proportions of many endemic plant and animal taxa specifically adapted to cool temperatures and humid—moist conditions. Although the TMCFs are less diverse than the lowland forests, when their exceptionally high levels of regional endemism are considered, their collective species diversity probably exceeds that of any other forest type (White 1983; Hamilton et al. 1995; Still et al. 1999; Wikramanayake et al. 2002; WWF 2007).

Confinement of ground-dwelling arthropods of TMCFs to narrow altitudinal belts and their adaptations to exist in specific habitat conditions make these arthropods sensitive to habitat loss and fragmentation (Olson 1994; Brühl et al. 1999; Anu and Sabu 2007). Because ground-dwelling arthropods are better habitat predictors than arboreal arthropods, any conservation strategy should emphasize the distributional patterns of invertebrates as a basis for designing effective conservation strategies for TMCFs (Koen and Crowe 1987; Desender et al. 1991; Olson 1994). Little is known about the grounddwelling arthropod diversity in Asian TMCFs since a majority of the studies refer to vertebrates (Dowsett 1985; Rice 1988; Brooks et al. 1999; Shanker and Sukumar 1999) and plants (Foster 2001; Bussmann 2001; Thomas and Palmer 2007; Giriraj et al. 2008). Given this context, robust quantitative and qualitative assessments of the ecology and distribution of ground-dwelling arthropods in TMCFs are necessary to assess the effectiveness of conservation efforts practiced in tropical montane ecosystems.

Sampling of arthropods from high-altitude, wet terrestrial habitats is always hindered by practical difficulties, especially when random and quantitative samples are necessary. In particular, the unique and inherent bias of every such sampling method either excludes or underestimates abundances of some groups and renders interpretations difficult. Behavioural differences among faunal elements and the characteristics of the habitat where sampling is to be done, strongly influence the sampling techniques (Melbourne 1999; Southwood and Henderson 2000; Woodcock 2005). Unique habitat conditions in TMCFs with persistent cloud cover, cloud stripping, fog precipitation, low levels of solar radiation, continuously wet substrates and waterlogged forest floor, high relative humidity and low evaporation rates, cool temperature, and humid and moist environment limit the conditions for grounddwelling arthropods (Olson 1994; Brühl et al. 1999; Bruijnzeel 2001; Holder 2006). Moreover, the innate biases in the pitfall trap, Berlese funnel and Winkler extractor for surveying ground-dwelling arthropods, make it difficult to choose the most appropriate method to sample the ground-dwelling arthropods in tropical montane forests (Sabu and Shiju 2010).

Spatially and temporarily restricted densitybased quadrat sampling techniques (Berlese and Winkler extraction methods) may fail to capture many active groups (Edwards 1991; Spence and Niemelä 1994; Bestelmeyer et al. 2000; Robertson 2007). Pitfall trap is inefficient in capturing either the ground dwelling sedentary terrestrial arthropods or those which disseminate by flying and do not perform as well as quadrat extraction methods in sampling terrestrial arthropods from forest ecosystems with a well-developed litter layer (Fisher 1999; Woodcock 2005). While many papers consider the relative merits of modifying a particular sampling method focusing on particular taxa (Bremner 1990; Topping and Sunderland 1992; Holland et al. 1999; Ward et al. 2001; Work et al. 2002; Brennan et al. 2005; Krell et al. 2005), no attempts have been made to critically evaluate and quantitatively compare the extraction efficiency of the methods for sampling ground dwelling arthropods in Asian TMCFs.

**Figure 1.**
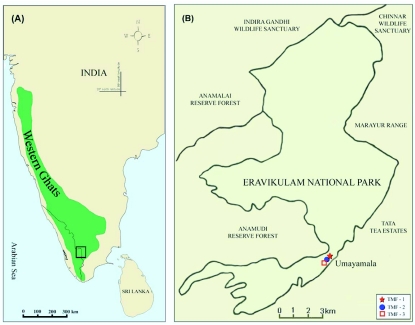
(A) Map of south-western India showing the location of the Western Ghats and (B) study site in the Eravikulam National Park. High quality figures are available online.

Our goal was to compare the efficiency of the pitfall trap, Berlese funnel and Winkler extractor in a TMCF in the Western Ghats, a global hotspot of biodiversity in south-western India, by seeking answers to the following questions:

What are the relative trapping efficiencies of these three widely used trapping methods, measured in terms of abundance and frequency of occurrence of ground dwelling arthropods, to obtain baseline information as rapidly as possible?
Which taxa are most likely collected in a baseline study using the three sampling methods?
Which taxa are the best collected by specific-trapping methods?

## Materials and Methods

### Study area

**Figure 2.**
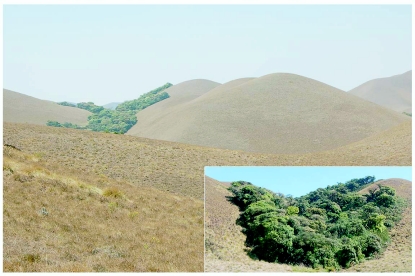
Tropical Montane Forest (TMCF) patch amidst grass land in Eravikulam National Park of the Western Ghats. High quality figures are available online.

The study site was at the Eravikulam National Park (ENP) (10° 10′ - 10° 20′ N; 77° 0′ - 77° 10′ E; 97 km^2^; Idukki District, Kerala State) (Figure 1), on the western slope of southwestern Ghats montane rain forests ecoregion (IMO 151) at 1400–2694 m (WWF 2001; Wikramanayake et al. 2002; Kerala Forests and Wildlife 2008). Patches of TMCFs surrounded by extensive grasslands (Southern Montane Wet Grasslands) prevail in the high altitudes at ENP (Figure 2). In southern India, the TMCFs and montane wet grasslands, generally found at an altitude above 1800 m in the Western Ghats are commonly known as shola forests and shola grasslands (Ranganathan 1938; Nair and Khanduri 2001). Annual climate features include temperature 17–20° C; RH 40–90%; mean annual rainfall 1300 mm; mean rainfall of southwest monsoon (June–August) 260 mm, northeast monsoon (September–November) 105 mm, presummer (December–February) 20 mm; summer (March–May) 50 mm (KDHP 2005– 07).

### Data collection

Because of the ca 100 km^2^ area, three patches named TMCF 1, TMCF 2, and TMCF 3 (each of 1–2 ha) on the east-facing hill slopes at 2200 m asl on the eastern side of ENP were selected for sampling. These TMCFs were at 500 m distance from each other. Three parallel 100 m line transects, separated by a 10 m inter-transect distances, were constructed west—easterly in each patch at a distance of 10 m from the forest edge. The mid-transect was used for pitfall traps and those on either side of the mid-transect were used for collecting litter samples (hereafter referred as litter sample collection locations) for Berlese and Winkler methods. A 25 m inter-trap distance between two consecutive pitfall traps and litter sample collection locations was maintained following Digweed et al. (1995). Sampling was done on three occasions: the first in the last week of September (20 September 2006; north—east monsoon), the second in the last week of January (22 January 2007; pre-summer), and the last in the fourth week of May (20 May 2007; summer). No sampling was done during south—west monsoon time because the heavy rain leaves forest floor water logged, moreover, road access to the site has always been nearly completely obstructed. Litter sample collection spots during the second (22 January 2007) and third sampling (20 May 2007) occasions were selected at a location 2 m ahead of the spot selected for collecting litter during the first occasion (20 September 2006) to avoid possible under-sampling of arthropods by repeated collection of litter from the same location. Collection of litter samples for Berlese and Winkler methods and placement of pitfall traps were done between 09:30 to 11:00 on the first day of each sampling occasion. All pitfall trapped materials were retrieved after 24 h on the following day between 09:30 to 11:00. Forty five samples (15 samples × 3 methods) were collected during each sampling occasion.

Litter samples from Berlese and Winkler methods were obtained by placing a 50 × 50 cm^2^ wooden frame on the forest floor and by collecting the leaves, litter, and loose humus that occurred within the frame (Frith and Frith 1990). Samples for extraction were sieved in a 1.5 cm mesh wire sieve, and the litter and sieved samples were saved in large cloth bags preventing possible escape of any arthropod. The litter thus collected included the upper organic litter layer and the loose humus layer. No underlying compact soil was obtained. Litter samples for Berlese and Winkler methods were transported to the laboratory in individual cloth bags. Care was taken ensuring litter samples were processed within 24 h and were not exposed to extreme changes in temperature, dryness, and humidity.

**Berlese funnel.** Fauna were extracted with a Berlese funnel apparatus (funnels were 30.5 cm in diameter, 35.6 cm height, with 4–6 mm mesh screens, fitted with 25 w tungsten—filament lamps) over Ehrlen-Meyer flasks containing into 70% alcohol placed at the end of the funnel stems over five days.

**Winkler extractor.** Litter samples were placed in coarse-mesh bags, which were suspended inside a large closed cloth bag suspended over a collection bottle 100 ml containing 50 ml of 75% ethanol (Besuchet et al. 1987). The litter and soil were left to dry at room temperature for five days. The litter material was gently mixed every day to ensure that the fauna remained active and to improve their chances of dropping into the collection bottle (Besuchet et al. 1987; Parr and Chown 2001).

**Pitfall trap.** Each trap consisted of a black plastic bowl (21 cm diameter, 15 cm depth) buried up to its rim in soil and partly filled with 50 ml of propylene glycol. Each trap was roofed over with a transparent sheet supported on iron pegs to prevent entry of rainwater and falling leaves and debris, which may facilitate escape of trapped fauna; such a system operated for 24 h continuously to avoid possible bias in captures arising from diurnal activity variation of fauna (Mommertz et al. 1996).

Trapped fauna were distinguished by observing the arthropods in a dissecting microscope (Labomed CZ 70; Labomed India Ltd; Ambala, India), and were identified up to superorder/order/family levels following Borror et al. (1989) and by comparing them with the type specimens in the museum collections at Entomological collections of St. Joseph's College, Devagiri, Calicut.

The frequency of occurrence and abundance of taxa in each trapping method was recorded. Frequency of occurrence means the frequency of collection (i.e. proportion of traps in which each taxon was found) and frequency of abundance means the total number of individuals of a particular taxon per sample in each trapping method. All determined specimens were deposited in the Entomological collections of St. Joseph's College, Devagiri, Calicut.

Hymenopteran taxa other than Formicidae and Chalcidae are collectively referred as ‘Other Hymenoptera’. Taxa at >25% frequency in any of one of the sampling method was considered ‘major’, and those at <25% frequency were considered ‘minor’. The sampling method, which trapped >25% frequency of a particular taxon, was deemed ‘reasonably effective’ in sampling of that particular taxon.

The sampling effort was calculated based on the time spent for field placement of traps and retrieval after 24 h for pitfall traps; for the Berlese and Winkler methods, collection of litter samples extraction and sorting of faunal groups was calculated during the pre-summer period. The pre-summer period was selected for cost estimation because in this period the highest ground dwelling arthropod abundance had been noted in the moist Western Ghats (Anu et al. 2009; Anu 2006; Vineesh 2007). The length of time needed for overall sampling was estimated by both excluding and including the 5 days of time taken for Winkler/Berlese funnel extraction of fauna in the laboratory. However the time spent on the extraction of fauna in the laboratory did not include Winkler/Berlese methods because they did not need continual attention. In contrast, with pitfall traps the sampling person had to wait for 24 h to retrieve the samples.

### Data analysis

Differences in the frequency of arthropod taxon among sampling methods (abundance data with median and inter quartiles, low abundance, and total absence of some taxa) rendered comparisons through the application of common parametric statistics inappropriate. The Winkler extraction method emphasized seeking differences in the frequency of occurrence of arthropod types more and testing for differences in the mean number of arthropod types following Prasifka et al. (2007) less. Higher frequency of taxa obtained (more often through a particular method than by the other two) rendered this reliable.

**Table 1.**
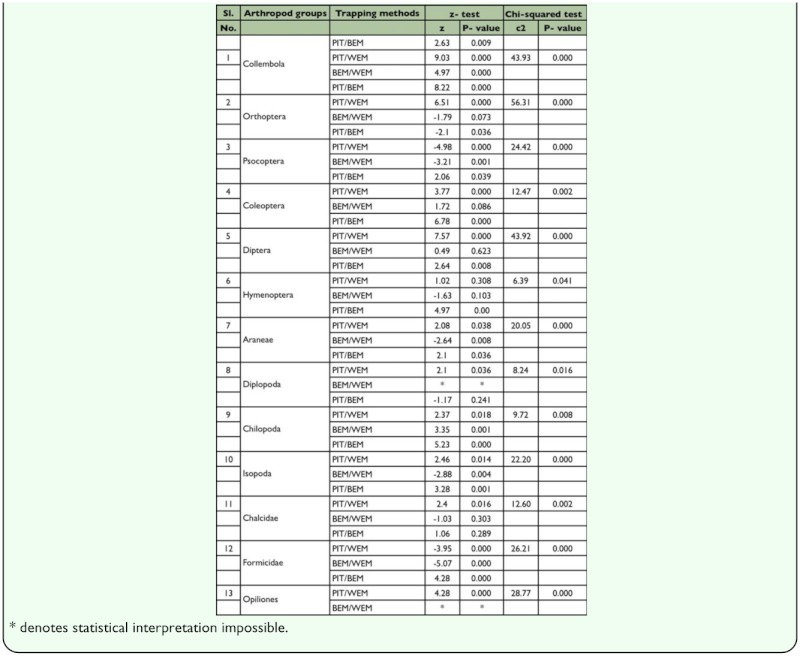
Results of Chi-squared test and z- test on the variation in the frequency of collection for ground dwelling arthropods using PIT, BEM and WEM

To summarize, arthropod captures by trap type, median, and inter quartiles derived from individual trap were calculated for each arthropod group. To test for differences in the frequency with which particular arthropod taxa were collected by the three trap types, 2 × 3 contingency tables categorized each trap as either successful (one or more individuals collected) or unsuccessful (zero individuals collected); the differences were assessed with χ^2^tests. Significant χ^2^ values indicated an effect of trap type on the proportion of samples containing one or more individuals of an arthropod taxon (Prasifka et al. 2007). Trap—wise differences in the capture efficiency of individual taxa among the three trap types were assessed with two sample z tests. Univariate comparisons through Kruskal—Wallis H tests were used to evaluate the significance level of trap-wise differences among medians in faunal abundance. When significant differences were found, a Mann— Whitney U—test was applied to determine which pairs of methods differed significantly (Weiss 2007).

Mean and standard deviation of the length of time required for sampling with each trap type were calculated. Trap—wise differences in the length of time needed to collect, extract, and sort samples were assessed with ANOVA, and when the differences were significant pairwise analyses were done with Tukey—Kramer test. All the analyses were done using MegaStat Version 10.0 (Orris 2005).

**Figure 3.**
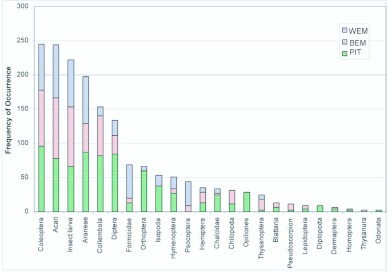
Percentage of frequency of ground dwelling arthropods collected from pitfall traps, Berlese and Winkler extraction methods. High quality figures are available online.

## Results

Altogether, fauna from 135 samples with 45 from each method were available for data analysis. From the three methods tested, 5275 individuals belonging to 24 arthropod taxa were collected. These arthropod taxa could be broadly divided into a major group of 12 taxa with > 25% of frequency in any one method and a minor group of 12 taxa with <25% of frequency (Figure 3). Based on the differences in capture among the tested trapping methods, arthropod taxa could be further divided into a group of 13 taxa comprising 10 major and three minor groups. These groups showed significant differences in capture among the tested trapping methods (Table 1), and another group of 11 taxa comprising two major and nine minor groups with no difference in capture among the tested trapping methods. Based on the frequency of occurrence of fauna, 22/24 taxa were obtained in pitfall traps, 19/24 in Berlese funnels, and 14/24 in Winkler extractors (Figure 3).

The proportionate distribution of dominant taxa in the collections from Winkler extractors was in the following sequence: Acari (78%) > Araneae (69%) = larvae of insects (69%) > Formicidae (49%) > Psocoptera (36%) > Coleoptera (30%). The highest frequency of occurrence was recorded for taxa belonging to Psocoptera and Formicidae, and an equivalent level of frequency of occurrence as those from the Berlese method for seven taxa: Orthoptera, Coleoptera, Diptera, other Hymenoptera, Chalcidae, larvae of insects, and Acari. A comparison of the captures from the Berlese and Winkler methods showed that the Berlese funnels recorded the highest frequency for Collembola and Chilopoda, whereas the Winkler extractors recorded the highest frequency for Formicidae, Psocoptera, Isopoda, and Araneae (Figure 3).

The dominance pattern of major taxa in Berlese extraction method was ‘Acari (89%) > larvae of insects (87%) > Collembola (58%) > Araneae (42%) > Coleoptera (37%)’. Berlese funnels recorded an equivalent level of frequency of occurrence as the Winkler extractors for seven taxa, and the same frequency of occurrence as that obtained in pitfall trap for Formicidae (Figure 3).

Proportionate capture of the dominant taxa in pitfall trap was ‘Araneae (87%) > Diptera (84%) > Collembola (82%) > Acari (78%) > larvae of insects (67%) > Orthoptera (60%) > Coleoptera (43%)’. For the Collembola, Orthoptera, Coleoptera, Diptera, Hymenoptera, Araneae, Diplopoda, Isopoda, Chalcidae, and Opiliones, the pitfall trap yielded the highest frequency and for categories such as larvae of insects and Acari, a similar frequency as that obtained in the other two methods. Opiliones and Diplopoda were groups unique to the pitfall trap, and were recorded at a high frequency (with >50% more than what was recorded in the other two methods) in pitfall traps (Figure 3).

**Table 2.**
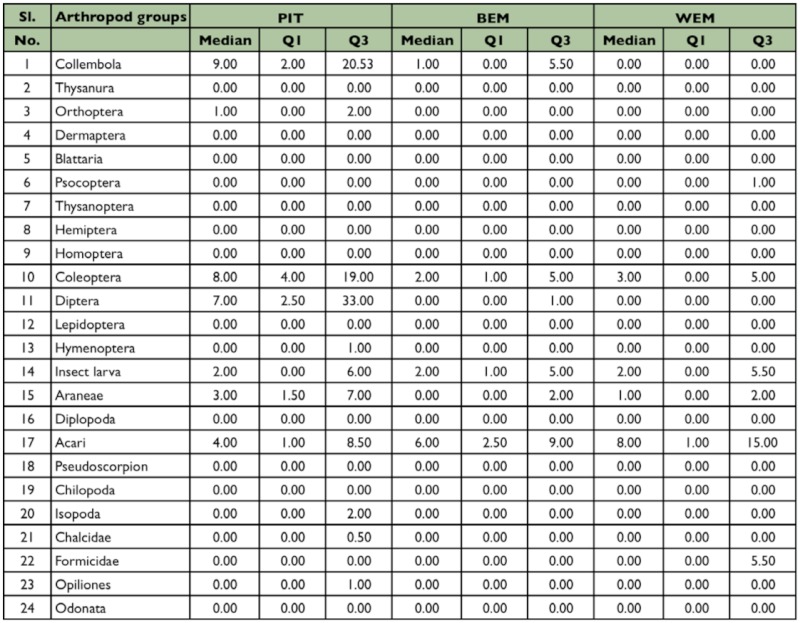
Median and inter-quartiles (Q1 and Q3) of abundance of ground dwelling arthropods collected from PIT, BEM and WEM.

In brief, pitfall traps recorded the highest frequency for 10 out of 13 taxa (eight major and two minor taxa), Winkler extractors for 2 out of 13 and Berlese funnels for none (0 out of 13). All three methods recorded same level of frequency of occurrence for the larvae of insects and Acari. Among the quadrat methods, Berlese funnels were effective for 7 out of 13 groups (Berlese funnels were ineffective for two pitfall trap method unique groups: Diplopoda and Opiliones; and the four groups: Psocoptera, Araneae, Isopoda and Formicidae, for which Winkler extractor is superior); and the Winkler extractor was effective for 9 out of 13 groups (Winkler extractor was ineffective for the pitfall trap unique taxa (Diplopoda and Opiliones) and Collembola and Chilopoda for which Berlese funnel is superior) (Figure 3).

**Figure 4.**
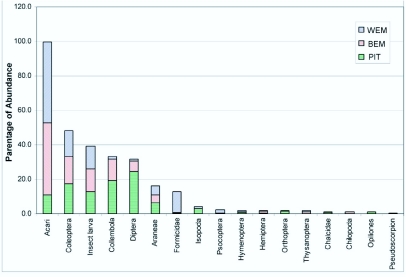
Percentage of abundance of ground dwelling arthropods collected from pitfall traps, Berlese and Winkler extraction methods (groups <0.5% is excluded). High quality figures are available online.

Abundance data for the ground-dwelling arthropods (median ± inter quartiles) in the three sampling methods have been summarized in Table 2. Pitfall traps recorded the highest abundance of 10 out of the 13 arthropod taxa among the three tested methods (Figure 4). Berlese funnels recorded the highest abundance for Chilopoda, and Winkler extractors recorded the highest abundance for Psocoptera and Formicidae. A comparison of data from Berlese and Winkler methods showed that Berlese funnels recorded the highest abundance for Collembola and Chilopoda, and Winkler extractors for Psocoptera and Formicidae (Table 3).

Comparison of cost in terms of length of time required to sample (= collect, sort, and identify a sample) ground-dwelling arthropods showed significant differences among the three trap types (Table 4). When the time taken for extraction of fauna in the Berlese/Winkler methods was excluded, pitfall traps required the longest time for collection and overall sampling. Of the two quadrate methods, Winkler extractors required the lowest duration for sorting and overall sampling and Berlese funnels required the lowest duration for sample collection and preparation for extraction.

## Discussion

**Table 3.**
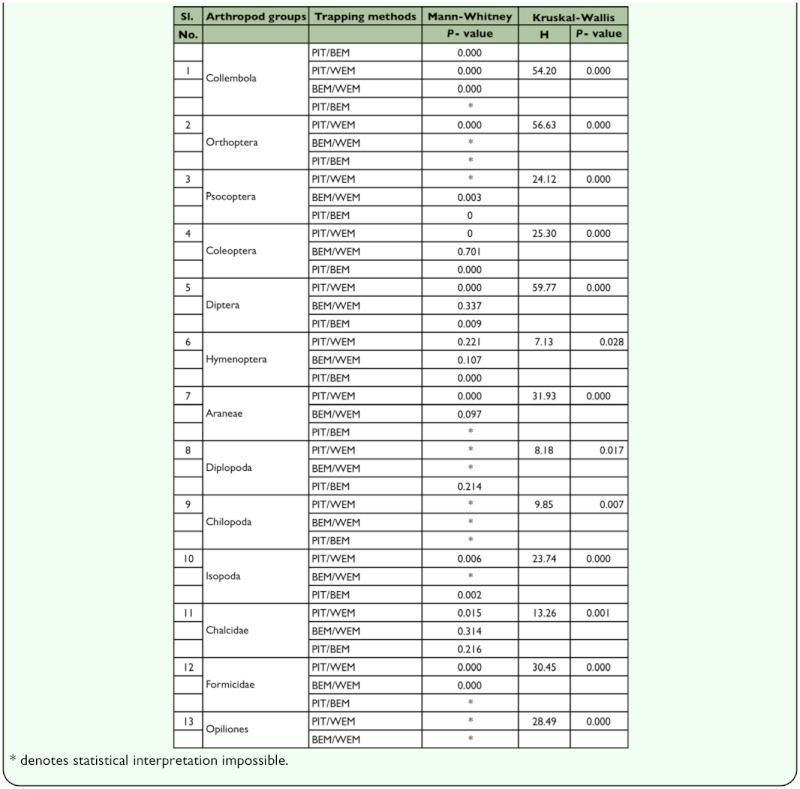
Results of Kruskal-Wallis and Mann-Whitney tests on the variation in the abundance of collection for ground dwelling arthropods using PIT, BEM and WEM.

All methods used for sampling grounddwelling arthropods in TMCFs produced biased data. Density-based quadrat estimators (Berlese and Winkler methods) sampled less of a few taxa, and the activity-based pitfall traps sampled less of a few groups and sampled more of many other groups. Among the three methods, Winkler method was most efficient for exhaustive quantitative data and the pitfall trap was most efficient for qualitative data of most ground-dwelling arthropods in TMCFs. Group and trap-specific differences noted in the present study supports the earlier findings (Edwards 1991; Standen 2000) that no single method is the best for all taxa of ground-dwelling arthropods, and it may be necessary to efficiently combine two or more methods (of course governed by the aims of the study).

A pronounced difference occurred among the three tested sampling methods. Pitfall traps yielded the maximal capture (both frequency and abundance) of 12 out of 24 taxa, followed by the Winkler method for 02 out of 24 taxa, and the Berlese method for 0 out of 24 taxa; and for larvae of insects and Acari all the methods are equally effective. The Berlese method proved the least effective among the three methods for any taxa. Pitfall traps become indispensable for Diplopoda and Opiliones and for Orthoptera and Diptera with exceptionally high abundance and frequency of capture. These percentages (effective capture of 50% of the whole taxa) indicate that the pitfall trap is the most useful arthropod collection method for ecological studies of ground-dwelling arthropods, when compared with Berlese and Winkler methods. Non-significant differences in the capture of minor taxa (9 out of 24) among the different trap types are difficult to interpret because of their low frequency of occurrence and abundance possibly related to the low population densities of these taxa in the wet forests of the Western Ghats (Anu 2006; Vineesh 2007; Anu et al. 2009).

**Table 4.**
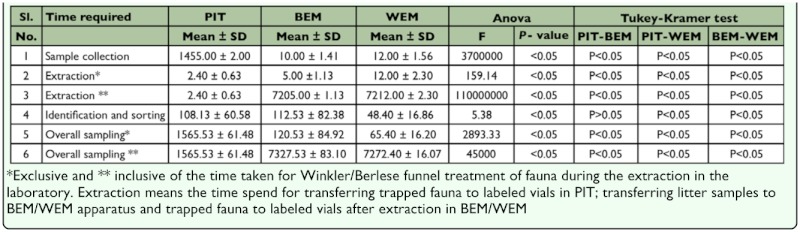
Comparison of the cost in terms of the overall sampling time (Mean ± SD in minutes) needed to collect, sort and identify a sample of ground dwelling arthropods employing PIT, BEM and WEM and significance levels of statistical analysis in a TMF site in the Western Ghats.

However, a strong bias was apparent in the samples obtained with pitfall traps compared with the Berlese and Winkler methods. Pitfall trap traps captured more taxa of surface-active invertebrates: Orthoptera, Diptera, Araneae, Collembola, Coleoptera (with more of Staphylinidae), other Hymenoptera, Chilopoda, Diplopoda, and Opiliones (Dennis et al. 1997; Bignell et al. 2000; Prasifka et al. 2007) in comparison to their relatively low frequency of capture in Berlese and Winkler methods. The Formicidae were less frequently caught in pitfall trap traps, which is not surprising because of their low occurrence, their cryptic nature and underground nesting habits in TMCFs (Brühl et al. 1999; Anu and Sabu 2007; Vineesh et al. 2007) and the inefficacy of pitfall trap traps in sampling Formicidae in wet-forest habitats (Fisher 1999). The prominent taxa in the captures of the Berlese and Winkler extraction methods, were the sedentary taxa that occurred in higher abundance in moisture and sheltered areas, including: Isopoda, Psocoptera, Formicidae, Collembola, and Coleoptera (with more of Curculionidae) in higher abundance than Orthoptera, Diptera, Araneae, Collembola, Coleoptera (with more of Staphylinidae), other Hymenoptera, Chilopoda, Diplopoda, and Opiliones. Such well known predisposition of pitfall trap traps towards surface-active invertebrates and the difficulty in comparison of the data with Berlese and Winkler methods in quantitative estimation (Topping and Sunderland 1992; Spence and Niemelä 1994; Oliver and Beattie 1996; Work et al. 2002; Woodcock 2005) make the density—based estimators (Berlese and Winkler methods), which measure populations in numbers of arthropods/unit area, the only alternative for multitaxa quantitative ecological studies of ground— dwelling arthropods.

In addition to the above limitations, pitfall trap traps necessitated a second field visit to the high-altitude TMCFs to retrieve the traps involving additional expenditure, time loss, and practical difficulties in protected forests with restricted access. Moreover, multiple chances of wildlife disturbing the field-placed traps, and inclement weather, especially strong winds, affecting the sampling effort in TMCFs, also exist. Such a situation would generally leave the researcher in suspense on the success of the collection efforts until the second trip and would make collection of samples for pitfall trap more laborious, costly, and unreliable than Berlese and Winkler methods. Since extraction of fauna with Berlese/Winkler methods apparatus is a laboratory based, passive activity and the researcher could utilize the time usefully for other activities, it was considered reasonable in the present study to exclude the length of time taken for faunal extraction by the Berlese/Winkler methods from the overall sampling time and from comparative analysis of cost. When the time taken for extraction of fauna with Berlese/Winkler methods apparatus is excluded, pitfall traps become less efficient than Berlese/Winkler methods for overall sampling. By contrast, the pitfall trap has one clear advantage over the spatially and temporarily limited quadrat sampling methods (Berlese and Winkler methods) as it enables collection of nocturnal and diurnal guilds of taxa.

This limits the choice of methods to two density based quadrat estimation methods (Berlese and Winkler methods). Recent studies in the moist deciduous forests of the Western Ghats showed that the Berlese method was a more efficient alternative method for exhaustive extraction of grounddwelling arthropods than Winkler extraction method (Sabu and Shiju 2010). However, contrary to expectations, in the present study the Winkler method was found superior to the Berlese method in tropical montane cloud forests. In comparison with Winkler, the Berlese method underestimated the frequency and abundance of four major taxa: Psocoptera, Araneae, Formicidae, and Isopoda in southern Indian TMCF conditions, but performed well for capturing Collembola.

Lower occurrence of Formicidae, Isopoda, Psocoptera, and Araneae using the Berlese method compared with the Winkler method could be due to the factors of heat and desiccation that are used for the Berlese method, which is a weakness in extracting fauna from moist forests (Bestelmeyer et al. 2000). Ground—dwelling arthropods of cool, wet, and moist TMCFs with a closed canopy are mostly ground nesting and are never exposed to dry conditions, even in summer. Exposure of such ground dwelling fauna adapted for the cool, moist habitat conditions to the dry conditions in Berlese method are likely to lead to their death from desiccation before dropping into the collection jars. We see no other reasons, as our experience from moist forests showed that Formicidae, Psocoptera, and Araneae were effectively sampled by Berlese and Winkler methods. This brings to focus the heat sensitivity of high elevation ground-dwelling arthropod fauna of TMCFs and the ineffectiveness of the heat-driven Berlese method in sampling them.

The presence of the two pitfall—trap unique groups, Opiliones and Diplopoda, and the low abundance of Orthoptera, Diptera, and Collembola indicate that use of the Winkler method alone will lead to underestimation of these taxa, thus leaving the researcher with two choices: (1) ignore the under-represented groups (Dennis et al. 1997); or (2) extract the five under sampled taxa (Collembola, Orthoptera, Opiliones, Diplopoda, and Chilopoda) from the Winkler litter samples with the floatation technique (Edwards 1991). Lower representation of Collembola in Winkler extraction method was attributed to the remarks of Besuchet et al. (1987) that the Winkler method is less suitable for the extraction of all taxa, and there is possibility of death of taxa with a narrow ecological tolerance before dropping into the collection bottles. More time and labour are required to sort out the fauna from the fallen debris and soil in laboratory, and the limited volume of quantitative information generated by the Berlese method compared with the Winkler method makes the Berlese method a less-efficient sampling method for ecological studies of ground-dwelling arthropod fauna in TMCFs.

To summarize, the trapping success of pitfall traps confirms the findings of Spence and Memelä (1994) that pitfall traps remain the most realistic way to survey large acreages where qualitative inventory and a comparison of species assemblages of ground-active arthropods is required. However, undersampling of the bottom-dwelling and moisture-preferring groups Formicidae and Psocoptera in pitfall traps, require the use of the Winkler method in TMCFs even if the aim of the study is purely qualitative inventory. For quantitative studies of ground-dwelling arthropods in TMCFs, the Winkler method is the best option. Nonetheless, TMCFs lie at various altitudes in the subtropical and tropical regions with distinctive regional patterns in climate, vegetation types, and faunal distribution patterns. As these inferences are based on the study in a high elevation TMCF in the Western Ghats, the recorded effectiveness of the Winkler method may not be appropriate for all TMCFs in other longitudinal grids.

## Conclusions

The relative frequency of occurrence and abundance of fauna were different with each of the three sampling methods. When cost and time constraints dictate limiting of grounddwelling arthropod sampling to one method, the Winkler extraction method is ideal for quantitative estimation and the pitfall trap is ideal for qualitative estimates in TMCFs. The low incidence of five taxa: Orthoptera, Diptera, Opiliones, Araneae, and Diplopoda collected by the Winkler method necessitates inclusion of a complementary floatation method, which would enable a comprehensive quantitative survey of ground-dwelling arthropods. Although pitfall traps tend to collect more of the ground-active species, its efficiency indicates that the pitfall trap is certainly the method of choice for an individual qualitative sampling method for most major taxa except the Formicidae and Psocoptera. For Formicidae and Psocoptera, the Winkler method is the best option. As a cost-effective individual quantitative sampling method, the Berlese method is suitable for collecting larvae of insects, and Acari, and Chilopoda in TMCFs, but is not suitable for ecological studies involving multiple arthropod groups or for other taxa.

## Abbreviations

**ENP**,: Eravikulam National Park;
**TMCF**,: tropical montane cloud forest
